# Dr. Levy on Inflammation of the Umbilical Arteries, as a Cause of Trismus Neonatorum

**Published:** 1842-05-28

**Authors:** 


*On Inflammation of the Umbilical Arteries, as a cause of Trismus Neonatorum.* By Dr. Levy.—This theory was originally promulgated by Colles, in 1818, but has since been opposed by Labatt and Elsässer, whilst it received additional confirmation from the researches of Busch of Berlin, in 1837. Dr. Levy has had more numerous opportunities of investigating this remarkable malady than usually falls to the lot of practitioners in other parts of Europe.

In 1838-9 he attended 22 cases in the Lying-in Institution in Copenhagen. Of these, 20 died and only 2 recovered. Of those that died, 15 were examined carefully after death, and in 14 there were the most decided marks of inflammation in the umbilical arteries. Dr. Levy has found that the principal seat of the inflammatory action is in that part of the umbilical arteries which lies along the urinary bladder. Inflammation is always present in the artery of both sides, though rarely in an equal degree.

Considerable variety was observed in the appearance of the internal coats of the artery, but externally its diameter was always increased, and the peritoneum surrounding it presented evident marks of inflammation; for in several cases this membrane was much injected with red blood, and, in three instances, adhered, by effusions of coagulable lymph, to the jejunum or omentum. On incising the arteries themselves their walls were found much thickened, and their dilated cavities contained more or less of a dark reddish brown or greenish puriform matter, which was always extremely fœtid. Upon the removal of this matter the coats of the arteries presented the following alterations:

1. Inflammatory discoloration, and inequality of surface of the tunica intima.

2. Thickening of the tunica intima, or of its subjacent cellular tissue. This spongy tumefaction was one of the most frequent alterations in the umbilical arteries.

3. Ulcerative destruction of the tunica intima, so that in several points this membrane was totally wanting. This appearance was often accompanied by spongy thickening of the subjacent cellular tissue noticed above. These ulcerations were in general superficial, but in two instances the destructive process had extended to the peritoneum. In another case the umbilical artery of the right side was completely perforated, and the spot where this had occurred was surrounded by a layer of thick ichorous matter, which here and there covered also the posterior wall of the bladder.

4. Softening and consequent rupture of the walls of the umbilical arteries. This occurred but once, viz. in that portion of the artery which lies on the superior fundus of the bladder. From this point almost to the navel both arteries were in a state of gelatinous softening, their different coats no longer to be distinguished. At the distance of about an inch from the navel, was an irregularly oval and somewhat lacerated opening in the walls of the artery, from whence about an ounce of ichorous-looking fluid had exuded into the cavity of the pelvis. Even the peritoneum covering the artery was here completely softened, and the whole bore no small resemblance to the well-known gelatinous softening of the coats of the stomach.

5. Gangrene of the umbilical arteries was but once observed; in a child of seven days old, which died forty-eight hours after the commencement of the disease. In the artery of the right side there was found pus, with great thickening of the portion running along the urinary bladder, where also a firm coagulum was discovered attached to the inflamed walls of the vessel. The left umbilical artery was nearly double the size of the right, and externally of a blueish-green colour. It contained a large quantity of greenish-black ichorous matter, mixed with shreds of the tunica intima, and which exhaled a most putrid and offensive odour.

Dr. Levy paid particular attention in all these cases to the state of the external umbilicus, in order to ascertain how far inflammation of the umbilical arteries can be inferred from the appearance of the navel during life. In four cases it was perfectly unchanged during the whole course of the disease; in ten other instances the surface was in like manner unaltered, but the fundus was more or less red, and filled with puriform fluid, which quickly re-appeared when removed, and, in general, shortly before death, the naval became of a greenish colour.

Dr. Levy then proceeds to examine the theory of Elsässer, who in twenty cases had always discovered after death marks of congestion, and in sixteen of these actual extravasation of blood into the spinal canal. But Dr. Levy believes these appearances to result chiefly from the actual violence necessarily employed in laying open the spinal canal, even in young children; and also perhaps from congestion, as the result of the convulsive disease.

Dr. Levy has twice observed suppuration in the umbilical arteries after death, where no trismus occurred during life.

He has succeeded in saving two patients by a leech or two applied to the umbilicus, with warm fomentations to the abdomen, and by anti-spasmodics given internally.—*Brit. and For. Med. Rev.*, from *Bibl. for Læger*, Sept. 1840.

